# Solving the explainable AI conundrum by bridging clinicians’ needs and developers’ goals

**DOI:** 10.1038/s41746-023-00837-4

**Published:** 2023-05-22

**Authors:** Nadine Bienefeld, Jens Michael Boss, Rahel Lüthy, Dominique Brodbeck, Jan Azzati, Mirco Blaser, Jan Willms, Emanuela Keller

**Affiliations:** 1 Department of Management, Technology, and Economics, ETH Zurich, Zürich, Switzerland; 2 Neurocritical Care Unit, Department of Neurosurgery and Institute of Intensive Care Medicine, Clinical Neuroscience Center, University Hospital Zurich and University of Zurich, Zürich, Switzerland; 3 Institute for Medical Engineering and Medical Informatics, School of Life Sciences FHNW, Muttenz, Switzerland

**Keywords:** Health care, Social sciences

## Abstract

Explainable artificial intelligence (XAI) has emerged as a promising solution for addressing the implementation challenges of AI/ML in healthcare. However, little is known about how developers and clinicians interpret XAI and what conflicting goals and requirements they may have. This paper presents the findings of a longitudinal multi-method study involving 112 developers and clinicians co-designing an XAI solution for a clinical decision support system. Our study identifies three key differences between developer and clinician mental models of XAI, including opposing goals (model interpretability vs. clinical plausibility), different sources of truth (data vs. patient), and the role of exploring new vs. exploiting old knowledge. Based on our findings, we propose design solutions that can help address the XAI conundrum in healthcare, including the use of causal inference models, personalized explanations, and ambidexterity between exploration and exploitation mindsets. Our study highlights the importance of considering the perspectives of both developers and clinicians in the design of XAI systems and provides practical recommendations for improving the effectiveness and usability of XAI in healthcare.

Publications on artificial intelligence (AI) and machine learning (ML) in medicine have quintupled in the last decade^1,2^. However, implementation of these systems into clinical practice lags behind due to a lack of trust and system explainability^2,3^. Solving the explainability conundrum in AI/ML (XAI)^4,5^ is considered the number one requirement for enabling trustful human-AI teaming in medicine^2,3,6^; yet, current efforts consisting of complex mathematical methodologies (e.g., ante-hoc or posthoc procedures^7^) are unlikely to increase clinicians’ trust and practical understanding^8^. Furthermore, it is still unclear if and how clinicians and system developers interpret XAI (differently), and whether designing such systems in healthcare is achievable or even desirable^4,5,8^.

This study aims to answer these questions by exploring clinicians’ and developers’ mental models of XAI. We questioned 112 clinicians (physicians and nurses) and developers (1 data scientist, 1 senior product designer & visualization expert, and 2 senior software engineers) working in the Neuro Intensive Care Unit (N-ICU) of a large University Hospital in Switzerland, as they engaged in the co-design of the DCIP—an ML-based clinical decision-support system (CDSS) to predict the onset of Delayed Cerebral Ischemia in patients with aneurysmal subarachnoid hemorrhage (aSAH) (see Methods). This approach gave rise to a framework of different mental models of clinicians and developers and five design recommendations that support the design of XAI in acute care medicine^9^ (see Fig. 3).

During the one-year-long DCIP design process, we conducted a survey, a focus group, and 11 interviews with N-ICU clinicians and developers. The results were continuously fed back into the design process improving the system over time^10^.

*Survey results*: To assess the user needs and intention to use the DCIP at the beginning of the design process, we surveyed a total of *n* = 95 clinicians (see Methods below & Supplementary Methods for full survey instrument). The results of scenario-based questions about how clinicians assessed the risk of DCI in a patient with or without the use of the DCIP (Q1-3) are displayed in Fig. 1. Clinicians’ preferences regarding alarm modalities (Q4) and DCIP location (Q5) are displayed in the Supplementary Figs. 1, 2.

**Fig. 1:**
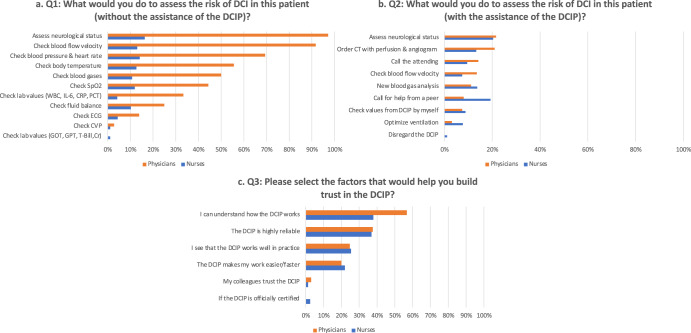
Clinician assessment of the risk of DCI in a patient with or without the use of the DCIP. **a**–**c** Frequencies (in %) of multiple response answers for survey Q 1–3. Responses from physicians are displayed in orange and from nurses in blue.

The means, standard deviations, and correlations between validated questions about user acceptance^11^ (Q6-8) and demographic variables are presented in the correlation matrix in Table 1.

**Table 1:** Descriptive statistics and correlations between survey variables.

	Variable	*M*	SD	1	2	3	4	5	6
1	Gender	0.37	0.48						
2	Professional Role	0.38	0.49	0.17					
3	Experience	16.17	11.45	−0.05	−0.56**				
4	Performance expectancy	5.29	0.95	0.22*	0.19	−0.02	(0.86)		
5	Effort expectancy	4.15	0.67	0.21*	0.27**	−0.14	0.58**	(0.81)	
6	Intention to use	6.37	0.78	0.11	0.02	0.04	0.38**	0.35**	(0.90)

*N* = 95; *p < 0.05, **p < 0.01, two-sided. Scale reliabilities (α) appear in parentheses along the diagonal. Gender: 0 = female; 1 = male. Professional Role: 0 = nurse; 1 = physician.

To test our hypothesis that DCIP performance and effort expectancy predict clinicians’ willingness to use the DCIP beyond gender, professional role, and experience, we conducted a two-stage hierarchical regression analysis with the intention to use the DCIP as the dependent variable. Assumptions of normality, linearity, and homoscedasticity were satisfied; sample size and collinearity statistics were within the accepted limits^12,13^. Gender, professional role, and experience revealed no significant results (*F* (3) = 0.53, *p* = 0.66; *R*^2^ = 1.7%), accounting only for 1.7% of the variance in intention to use the DCIP. Our predictor variables DCIP performance and effort expectancy however, revealed a significant change in *R*^2^ (*F* (2) = 8.55, *p* < 0.001), accounting for 15.8% of the variation in intention to use the DCIP, thus confirming our hypothesis.

*Focus group results:* The focus group revealed that the high-risk context and need for rapid decision-making did not allow for extensive system interactivity or the use of advanced analytics tools. As one attending stated: “When [the DCIP] gives me an elevated risk score, I must be able to see within minutes if [the results] make sense and whether I should order an emergency CT, administer an electrocyte transfusion, etc. We don’t have much time to waste by checking for more details. These [aSAH] patients, they can change [get worse] really quickly”.

*DCIP system user interface prototype:* Given clinicians’ generally high willingness to use the DCIP and expressed need for XAI (see survey results) coupled with the necessity for quick interpretations of model results (see focus group results), we developed a high-fidelity prototype of the DCIP user interface (UI) enabling a fast overview of DCIP results (see Fig. 2A–D) and interactive features requiring more time (see Fig. 2E, F).

**Fig. 2:**
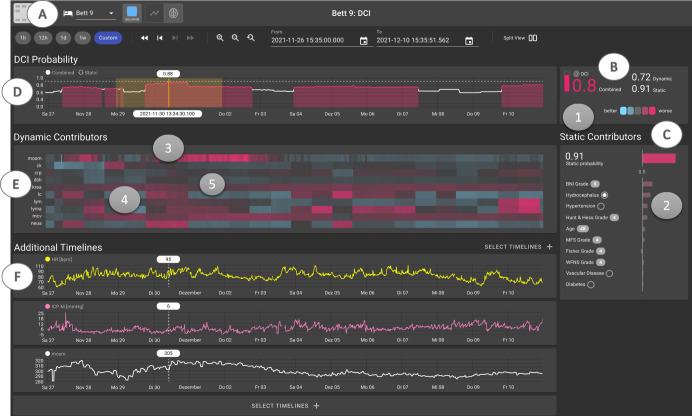
Screenshot of the DCIP system user interface prototype. The DCIP user interface aims to facilitate clinicians’ understanding of the ML model’s predictions with minimal time and effort. The header menu (**A**) displays information about the selected patient. In the overview frame (**B**), the current combined DCI risk score (0.8) is displayed, based on dynamic (0.72) and static (0.91) contributors (pink vs. blue hues indicating higher vs. lower risk). The static contributor view (**C**) displays Shapley values of static contributors assessed at the time of patient admission, including reference values for cohort-level evidence based on clinical norms such as the Barrow Neurological Institute Grading Scale (BNI), Hunt & Hess Grade, Modified Fisher Grade (MFS), Fisher Grade, and World Federation of Neurological Surgeons Grade (WFNS). The horizontal bar chart displays how the values below/above 0.5 decrease or increase the DCI risk score (in order of importance) The DCI probability frame (**D**) displays periods of high risk for DCI as colored areas under the curve, allowing clinicians to probe exact numeric values at each point in time. The solid line represents the combined risk fluctuating over time and the dashed line indicates the constant static probability (0.9). The Dynamic Contributors frame (**E**) displays a heatmap of Shapley values of dynamic contributors over time. Each heatmap lane shows how much a given signal (e.g., mOsm = Serum osmolality) contributes to the DCI risk at a given point in time (hover) and can be added (double-click) as an additional timeline displaying raw values below **(F**). Additional timelines can be added on demand to provide context for feature-level explanations (e.g., Heart Rate [bpm], intracranial pressure [mmHg], pupil reaction time). All timelines are in synch and can be zoomed/panned as desired. Individual points in time can be probed to reveal exact numeric values across all charts. For context information outside of the DCIP, clinicians can access the target patient’s complete health records (via the separate Electronic Health Record [EHR] system). Numbers 1–5 highlight the specific features referred to by interviewees when searching for model explanations (see Interview results).

*Interview results:* Our in-depth analysis of 117 pages of transcribed text using Grounded Theory^14^ revealed three conceptual themes (see Supplementary Fig. 3 for the data structure^15^ and Supplementary Table 1 for code descriptions and example quotations): The first theme *“Opposing Goals for XAI*” illustrates the contrast between developers’ and clinicians’ understanding of the fundamental purpose of XAI. Developers believed that “[clinicians] must be able to understand what the model is doing” and thus aimed to increase the interpretability of the model. They tried to achieve this, for example, by introducing Shapley values^16^ of static and dynamic risk contributors to help explain the local logic of the model at each point in time (see Fig. 2C, E, F). Clinicians, however, found this information unhelpful in increasing their understanding and trust in the system. When asked to make sense of the information displayed on the DCIP prototype, one attending physician stated: “Here [referring to Fig. 2B 1], is the overall risk score of 0.8 and here [referring to Fig. 2C 2], I see what contributes to the baseline risk. That makes sense. But this information over here [referring to Shapley values of dynamic contributors, Fig. 2E], I don’t need to know all this, I am not a mathematician. What I need to know is do these results [referring to Fig. 2B 1] make sense clinically […]. For instance, when I see that on November 30^th^ [Fig. 2E 3], the risk for DCI was high, then I want to know what we did that day; like did we do a CT scan, and did [the patient] actually develop a DCI? Also, if I administered Mannitol [medication to lower intracranial pressure^17^], would the risk go down then? These kinds of things I need to know to trust [the DCIP].” Hence, for clinicians, XAI was related to a system’s ability to demonstrate the plausibility of results within the clinical context. To establish that context, clinicians accessed additional patient-specific information (patient history, laboratory values, diagnostic tests, imaging, etc.) from existing EHR systems in parallel to using the DCIP. To facilitate such holistic patient assessments, future designs should aim for a complete integration of systems (which was not possible in our case due to a walled-garden design of the existing EHR system^18^).

The second theme “*Different Sources of Truth*” revealed that developers tended to rely on data as the most reliable source for decision-making because “the model chose the most relevant factors to make accurate predictions”. Clinicians, on the other hand, regarded these data-driven predictions as “only one piece of the puzzle” focusing more on other patient-specific sources of information. In particular, clinicians aimed to combine the data from the DCIP with non-quantifiable pieces of information such as physiological manifestations of neurological deficits (e.g., paralysis, aphasia, decreasing consciousness). As one attending physician explained, this important information could only be gathered via examining their patients directly: “[The DCIP] cannot see, hear, or touch the patient. For example, if [the patient] can’t move his arm anymore, this can be really important. [The DCIP] can’t see any of this [clinically relevant information gained from physical examinations]. Another resident physician pointed to the importance of this information by suggesting: “It would be great if we could enter [information from the physical examination] here [referring to Fig. 2F 4] so we could see how [the neurological deficits] combine with all the other data in the system.”

The third theme “*Exploration vs. Exploitation Mindset*” raised a fundamental question regarding the use and benefits of ML-based systems per se. On the part of developers, the major benefits and fundamental purpose of ML was to discover “unknown patterns in the data to learn something new” (i.e., exploring new knowledge). Clinicians, on the other hand, indicated that they would trust the system only if it relied on “established knowledge gained from clinical studies and evidence-based medicine” (i.e., exploiting old knowledge). These opposing mindsets became apparent throughout the design process, for instance when it came to deciding which biomarkers should be included in the model right up to the question of trusting the system. As one resident physician observed: “When I see here [referring to Fig. 2C 2] BNI grade equals 5, Hunt & Hess is 4 that makes sense because the relationship with DCI is well established [in evidence-based-medicine]. But when I see here [referring to Fig. 2F 4] it’s pink because the white blood cells are high, then it makes me wonder, can this be correct? I don’t know about this relationship [between leukocyte number and DCI], so maybe it could just be a random correlation.” Another attending physician reflected on this same issue: “If a certain biomarker pops up again and again, like here [referring to Fig. 2E 5] but there is no evidence in the literature on this biomarker [i.e., Creatinine] concerning DCI, it is hard to trust [the DCIP]. But what if the machine was indeed correct? Then we would discover a new biomarker and the potential for learning would be huge”.

Based on these insights and to better bridge the gap between clinicians’ and developers’ differing mental models about XAI, we defined the following framework together with five recommendations to improve the design of XAI in future ML-based prediction systems in healthcare (see Fig. 3).

**Fig. 3:**
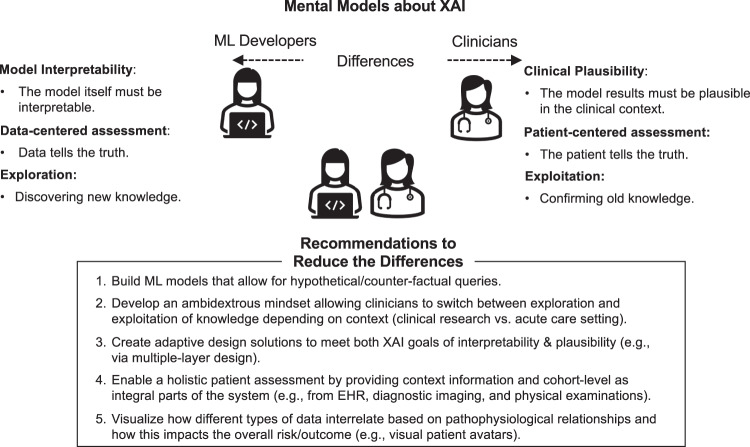
Framework of XAI mental model differences and recommendations how to reduce the differences (see textbox). ML Developers’ mental models (left) relate to model interpretability, a data-centered assessment, and an exploration mindset. Clinician’s mental models (right) aim for clinical plausibility, a patient-centered assessment, and an exploitation mindset.

The goal of this multi-method study was to explore how clinicians and developers interpret XAI (differently) and how the implementation of ML in healthcare can be facilitated by designing XAI solutions that consider the specific needs of each target group. For developers, increasing model interpretability is important to assess the reliability of the model and to eliminate bias^19,20^. For clinicians, on the other hand—although they were positive about using the DCIP and agreed with developers about the importance of XAI at the beginning of the study—when interacting with the DCIP prototype, established XAI solutions such as Shapely values of model contributors were unhelpful. Instead, they were looking for the clinical plausibility of model results. They did this by connecting model outputs with patient-specific context information gathered from EHR systems and by observing the manifestations of clinical symptoms in their patients. Recommendations for the design of future systems include the integration of information gained from physical examinations (e.g., via the Glasgow Coma Scale for Neurocritical Care^21^ or other clinical norms specific to each domain) as well as to find intuitive ways to visualize the complex pathophysiological interactions between different model contributors (e.g., patient avatars^22^).

Another point relates to increased system interactivity. For instance, it would be helpful for clinicians to receive cohort-level evidence from clustered patient data (e.g., to compare one patient to another within the same age group) or to probe the system via hypothetical/counterfactual questions. Such interactive designs have been proposed before (e.g., via WHAT-IF analyses^23,24^) and would likely address clinicians’ need for clinical plausibility. In our case, however, the usefulness of this approach was limited since probing the system with counterfactual questions and making changes (e.g., lowering creatine to improve kidney function), could misguide clinicians to wrongly assume that the therewith-associated reduction in the DCI risk score equals a reduction of the *actual* risk of a DCI occurring, which is not the case as our model was trained on input-target correlations only^25^. Moreover, in the fast-moving and high-risk context of the N-ICU, clinicians had very limited time for such interactions.

Finally, our findings regarding the exploration-exploitation mindset relate to the well-established literature on this binary concept in organizational learning and innovation management. There, the goal is to embrace ambidexterity, i.e., to switch between exploration and exploitation behaviors depending on the situational requirements^26^. A similar approach could help resolve the XAI conundrum in healthcare. Currently, developers’ and clinicians’ opposing mindsets and the associated differences regarding the goals and requirements of XAI hinder the successful implementation of ML in clinical settings. Like others before^27^, the developers in our study aimed to design the system to explore new knowledge, hence the need for increased model interpretability. Clinicians, on the other hand, entertained more of an exploitation mindset in that they were searching to confirm established knowledge in the context of time-sensitive decision-making. If the results seemed clinically plausible (i.e., in line with established knowledge), trust could be established without understanding how the model came to this conclusion. Besides, humans’ tendency to confirm rather than disconfirm prior knowledge is well-established in behavioral research and is often useful, especially in high-risk contexts such as medicine^28^. Nevertheless, this tendency also bears the risk of confirmation bias^29^ and the probability of missing out on the major benefits of ML, i.e., gaining new insights from big data and increased decision-making speed^2^. This conundrum was illustrated also in a recent study by Henry and colleagues^30^: Alerts for early warning of sepsis had to be purposefully delayed until clinical symptoms appeared in patients because clinicians trusted the system only then. To resolve this conundrum, we recommend designing XAI systems that meet both goals: the ability to explore new knowledge e.g., for research purposes, and to exploit existing knowledge in high-risk and time-critical contexts where model results must be validated quickly against established clinical standards^4,30,31^. Teaching clinicians how to switch between exploration and exploitation mindsets can help them adapt system interactions depending on different use cases and levels of expertise.

The major limitation of this study is that it relies on data from a single hospital and a single ML system. While access to a broader sample of ICU clinicians was not possible amid the COVID-19 pandemic, this study benefitted from the unique opportunity of gathering rich multi-method and longitudinal data from a fairly large sample of clinicians and developers as they co-developed the system in a real clinical context. Today, as ML in healthcare bears great potential but faces significant implementation hurdles^9^, this study shows ways to overcome them. Future studies on XAI in healthcare should consider the proposed design recommendations regarding the combination of model interpretability and clinical plausibility, increased system interactivity, and the importance of causal ML approaches allowing for hypothetical/counterfactual queries^23,25,32^. Furthermore, by reducing the differences between the mental models of developers and clinicians and adopting an ambidextrous mindset of exploration and exploitation, the design and application of XAI in healthcare can hopefully be improved for the benefit of all.

## Methods

In this multi-method study from October 2020 until December 2021, the research team conducted (1) an online survey with *n* = 95 ICU physicians and nurses, (2) a focus group with *n* = 3 clinicians and *n* = 3 developers, and (3) *n* = 11 interviews with clinicians and developers whom all participated in the co-design process of the DCIP. The study was approved by the Local Ethics Committee at ETH Zürich (No. EK 2019-N-190). Informed consent including the possibility to opt out of the study at any point and with no personal ramifications was obtained from all participants before data collection (in writing and/or by explicit agreement).

### Setting

This study was conducted at the N-ICU, University Hospital Zurich, a 12-bed ICU, treating about 1200 patients per year, mostly with severe ischemic and hemorrhagic stroke including aSAH, brain tumors, and epilepsy.

### System description

The DCIP system was based on a classifier trained to predict whether a specific patient will develop a DCI event during the subsequent 48 h. The development dataset consisted of 60 different factors including the patient’s medical history (age, gender, presence of diabetes, hypertension, cardiovascular disease), clinical presentation (GCS, Hydrocephalus, SAH severity scores such as Hunt & Hess, WFNS, BNI, MFS), as well as 30 different laboratory results, and 15 blood gas analysis (BGA) results. The laboratory and BGA values were measured regularly over the entire course of the patient’s stay in the ICU.

Behind the scenes, the DCIP system consisted of a static and a dynamic ML model, the former considering the parameters known at the time of patient admission to the ICU and the latter focusing on laboratory and blood gas analysis results, thus constantly adapting to a patient’s condition over time. For model training, time-dependent patient data were aligned using the initial DCI event as an anchor to capture the dynamics leading to DCI onset (c.f., Megjhani et al., 2020^33^). The output scores of the static and dynamic models were finally combined via a voting step (i.e., the final score is the average of the individual sub-models).

The different models were developed and cross-validated using data from 143 aSAH patients (48% with DCI event) treated in the neurosurgical intensive care unit of the University Hospital Zurich between 2016 and 2020. The software library “Scikit-Learn” 1.1.1 for Python 3.9.12 was used for modeling. To reduce the dimensionality of the data, a feature select step was integrated into the model training process using feature importance as a metric to select the 10 most important features for each model. Evaluation (nested cross-validation covering feature selection and hyperparameter tuning) of different tree ensembles showed that Extremely Randomized Trees^34^ performed best in the dataset. The training pipeline of the static model selected age, hydrocephalus, BNI, H&H, MFS, Fisher Scale, WFNS, cardiovascular disease, diabetes, and hypertension as features. The training pipeline of the dynamic model selected C-reactive protein, Creatine kinase, Creatinine, Interleukin-6, Leukocytes, Lymphocytes ratio, Middle corpuscular volume, Serum osmolality, and Neutrophils ratio. In the ROC analysis, the final voting model showed an AUC of 0.747 ± 0.094, while the dynamic and static models had an AUC of 0.729 ± 0.083 and 0.679 + 0.131, respectively^35^.

### Interface description

Based on the DCIP system, an interactive high-fidelity UI prototype was designed (see Fig. 2). The interface reproduces the DCIP system’s inputs (static & dynamic contributors) and outputs (probabilities) with pink vs. blue hues indicating a worsening or improvement of the situation. All charts update in real time, with time flowing from right to left. The top section (DCI Probability) visualizes all outputs of the system: on the right, the current combined risk (0.8) is displayed as a numeric value as well as a small bar visualization. The bar serves as a pink alarm indicator when the probability exceeds a certain threshold (e.g., 0.7). Analogously, colored areas under the curve on the left emphasize times of high risk. Static (0.91/ dashed line) and dynamic (0.72) probabilities are visualized separately to increase explainability of the combined risk score. The lower sections, for model explainability, visualize all inputs of the system: Shapley values^16^ “Static Contributors” are displayed as a waterfall plot (horizontal bar chart on the right), and “Dynamic Contributors” are visualized as a heatmap on the left. While each heatmap lane shows how a given signal contributes to the DCI risk, each signal’s raw values can be displayed as a timeline. Signals that are not direct contributors, but may still provide further explanation, can be added on demand. All visualizations are highly interactive: Timelines can be zoomed/panned as desired, and individual points in time can be probed to reveal exact numeric values across all charts.

### Data collection

The following types of data were collected:

*Survey:* 95 clinicians from the N-ICU participated in the online survey at the start of the design process (the response rate was high at 88.79%). To set the stage, clinicians received a video-based online instruction explaining the general purpose of the DCIP and how the system will be integrated as a plug-and-play application into the already existing ICU cockpit dashboard^35^. Participants were then presented with a patient scenario depicting an aSAH patient with ambiguous symptoms of an upcoming DCI, followed by scenario-based questions about (Q1) which actions they would take to assess the risk of DCI in the described patient *without* the assistance of the DCIP; (Q2) actions they would take to assess the risk of a DCI in this patient *with* the assistance of the DCIP; and (Q3) which factors would help them establish trust in the DCIP. Clinicians were also asked user experience (UX)-related questions such as alarm modalities (Q4) and their preference regarding the location of the DCIP (Q5) (see Supplementary Figs. 1, 2). The third part of the survey consisted of previously validated questions based on the Unified Theory of Technology Acceptance (UTATU)^11^ including performance expectancy (Q6), effort expectancy (Q7), and intention to use (Q8) the DCIP (Likert scale 1 = strongly disagree and 7 = strongly agree). Demographic variables included gender, professional role (nurses vs. physicians), and experience in years. The full survey instrument is provided in the Supplementary Method section.

*Focus group:* To specify the requirements analysis for the DCIP regarding XAI, we conducted a 90 min focus group with three developers and three clinicians online. The focus group protocol included questions about what XAI signified in the context of the DCIP and what kind of information was needed to increase explainability in the DCIP; which clinical tasks and practices preceded/proceeded interactions with the DCIP, and which special circumstances (time, risk) influenced the need for more (or fewer) model explanations.

*Interviews:* We conducted 11 interviews in total. First, we conducted four in-depth interviews exploring clinicians’ and developers’ mental models of XAI in the context of the DCIP in general (without interactions with the DCIP prototype). Second, we used the think-aloud method^36^ by asking seven clinicians to comment out loud on how they interpreted the various XAI features displayed on the DCIP prototype screen (see Fig. 2). Interviews lasted 46 min on average, were held online, and recorded via the Zoom video conferencing platform^37^. In line with Grounded Theory methodology, participants were purposefully selected to represent different levels of expertise. Data saturation was reached at the end of data collection when new interviews failed to generate new insights regarding the themes of interest^14^.

### Data analysis

Quantitative data from the survey were analyzed using descriptive statistics and linear regression modeling in IBM SPSS version 23. Audio and video recordings from the focus group and interviews were transcribed ad-verbatim and anonymized. Transcripts were methodically analyzed in a three-step procedure using Grounded Theory^14^ and the well-established Gioia methodology to ensure qualitative rigor in inductive research^15^. This approach is ideal for exploring new phenomena and building theory from rich qualitative data. Qualitative results are reported as per the standard published by the Academy of Medicine^38^. Two social scientists trained in qualitative research methodology analyzed the data iteratively and inductively by open-coding the complete set of materials from the transcribed text. To better illustrate the linkages between interview statements and the specific XAI features referred to by clinicians during DCIP prototype interactions, the data from interview transcripts were analyzed in line with the data from video files (i.e., coding while watching the respective video files). After agreeing on a coding scheme that best captured the diversity of the material, the transcripts and codes were analyzed again to identify second-order categories. First-order concepts included codes such as “Patient physiology is important” or “Data tells you only part of the story”. First-order codes were combined into second-order categories such as “The patient tells the truth”. Ultimately, second-order categories were merged hierarchically into three aggregate themes that best summarize the theoretical contribution (surprises and novelty) in the data: (1) “Opposing Goals for XAI”, (2) “Different Sources of Truth”, and (3) “Exploration vs. Exploitation Mindset” (see Supplementary Material Fig. 3 for the Gioia^15^ data structure and Table 1 for a complete list of codes, their definition and example statements).

### Reporting summary

Further information on research design is available in the Nature Research Reporting Summary linked to this article.

## Supplementary information

Reporting Summary

Supplementary Information

## Data Availability

Datasets that underpin the publication are deposited in the ETH Zurich Research Collection repository https://www.research-collection.ethz.ch/ and accessible by the Digital Object Identifier (DOI) 10.3929/ethz-b-000608438. The DOI issued to datasets in the repository can be included as part of a data citation in publications, allowing the datasets underpinning a publication to be identified and accessed. The full transcripts of the focus group and interviews as well as video files are not publicly available to minimize the risk of participant reidentification. Summaries of the interview contents and related metadata that support these findings are available from the corresponding author upon reasonable request.

## Code availability

The full set of code underlying the DCIP is not publically available but can be accessed from the corresponding author upon reasonable request. The software library “Scikit-Learn” 1.1.1 for Python 3.9.12 was used.
