# Supplementary material for: Solving the explainable AI conundrum by bridging clinicians’ needs and developers’ goals
Source: NPJ Digit Med. 2023 May 22;6:94. doi: 10.1038/s41746-023-00837-4 (PMC10202353; doi:10.1038/s41746-023-00837-4)
Supplement: Supplementary file 1 — Reporting Summary [file 41746_2023_837_MOESM1_ESM.pdf]

nature portfolio

Corresponding author(s): Bienefeld, Nadine

Last updated by author(s): 2023/04/20

Reporting Summary

Nature Portfolio wishes to improve the reproducibility of the work that we publish. This form provides structure for consistency and transparency in reporting. For further information on Nature Portfolio policies, see our Editorial Policies and the Editorial Policy Checklist.

Statistics

For all statistical analyses, confirm that the following items are present in the figure legend, table legend, main text, or Methods section.

n/a	Confirmed	
☐	☒	The exact sample size (*n*) for each experimental group/condition, given as a discrete number and unit of measurement
☐	☒	A statement on whether measurements were taken from distinct samples or whether the same sample was measured repeatedly
☐	☒	The statistical test(s) used AND whether they are one- or two-sided<br>*Only common tests should be described solely by name; describe more complex techniques in the Methods section.*
☐	☒	A description of all covariates tested
☐	☒	A description of any assumptions or corrections, such as tests of normality and adjustment for multiple comparisons
☐	☒	A full description of the statistical parameters including central tendency (e.g. means) or other basic estimates (e.g. regression coefficient) AND variation (e.g. standard deviation) or associated estimates of uncertainty (e.g. confidence intervals)
☐	☒	For null hypothesis testing, the test statistic (e.g. *F*, *t*, *r*) with confidence intervals, effect sizes, degrees of freedom and *P* value noted<br>*Give P values as exact values whenever suitable.*
☒	☐	For Bayesian analysis, information on the choice of priors and Markov chain Monte Carlo settings
☐	☒	For hierarchical and complex designs, identification of the appropriate level for tests and full reporting of outcomes
☐	☒	Estimates of effect sizes (e.g. Cohen's *d*, Pearson's *r*), indicating how they were calculated

Our web collection on statistics for biologists contains articles on many of the points above.

Software and code

Policy information about availability of computer code

Data collection	The software library "Scikit-Learn" 1.1.1 for Python 3.9.12 was used for modeling of the DCIP algorithm.
Data analysis	Quantitative data from the survey were analyzed using descriptive statistics and linear regression modeling in IBM SPSS version 23. Qualitative analysis was facilitated via MAXQDA software Version 20.

For manuscripts utilizing custom algorithms or software that are central to the research but not yet described in published literature, software must be made available to editors and reviewers. We strongly encourage code deposition in a community repository (e.g. GitHub). See the Nature Portfolio guidelines for submitting code & software for further information.

Data

Policy information about availability of data

All manuscripts must include a data availability statement. This statement should provide the following information, where applicable:
- Accession codes, unique identifiers, or web links for publicly available datasets
- A description of any restrictions on data availability
- For clinical datasets or third party data, please ensure that the statement adheres to our policy

Datasets that underpin the publication are deposited in the ETH Zurich Research Collection repository https://www.research-collection.ethz.ch/ and accessible by the Digital Object Identifier (DOI) 10.3929/ethz-b-000608438. The DOI issued to datasets in the repository can be included as part of a data citation in publications, allowing the datasets underpinning a publication to be identified and accessed. The full transcripts of the focus group and interviews as well as video files are not

publicly available to minimize the risk of participant reidentification. Summaries of the interview contents and related metadata that support these findings are available from the corresponding author upon reasonable request.

Human research participants

Policy information about studies involving human research participants and Sex and Gender in Research.

Reporting on sex and gender	Findings apply to gender male or female. Gender was self-reported. The quantitative results are reported separately for male and female gender.
Population characteristics	Social Science study. See above.
Recruitment	All clinical staff working at the N-ICU were invited to participate in the online survey at the start of the design process. Self-selection bias cannot be excluded. However, the response rate was high (88.79 %). In line with Grounded Theory methodology, participants for the qualitative parts of the study were purposefully selected to represent different levels of expertise.
Ethics oversight	The study was approved by the Local Ethics Committee ETH Zürich (No. EK 2019-N-190). The authors declare no competing financial or non-financial interests.

Note that full information on the approval of the study protocol must also be provided in the manuscript.

Field-specific reporting

Please select the one below that is the best fit for your research. If you are not sure, read the appropriate sections before making your selection.

☐ Life sciences ☒ Behavioural & social sciences ☐ Ecological, evolutionary & environmental sciences

For a reference copy of the document with all sections, see nature.com/documents/nr-reporting-summary-flat.pdf

Behavioural & social sciences study design

All studies must disclose on these points even when the disclosure is negative.

Study description	This is a longitudinal multi-method study consisting of an online survey, a focus group, and interviews.
Research sample	N = 112 developers and clinicians from the N-ICU participated in the study. Inclusion criteria was participation in the co-design of the DCIP. Self-selection bias cannot fully be excluded. However, the response rate was high (88.79 %). In line with Grounded Theory methodology, participants for the qualitative parts of the study were purposefully selected to represent different levels of expertise.
Sampling strategy	All clinical staff working at the N-ICU were invited to participate in the online survey at the start of the design process via email. Invitation were by the first author (via her secure ETH Zurich Email account). Each participant was provided with a random code to ensure anonymity. Sample size for the quantitative analyses was calculated using the Qualtrics sample size calculator for multiple linear regression. Data saturation was reached at the end of data collection when new interviews failed to generate novel insights regarding the themes of interest (Glaser & Strauss, 2017).
Data collection	Survey data were recorded via the secure survey platform Qualtrics. The first author had access to an institutional license to use this platform (via ETH Zurich). No researcher was present during quantitative data collection. All researchers but the first and last author were blind to the hypotheses / interest of exploration. Qualitative data from focus group and interviews were recorded via the Zoom video platform. Audio and video files were transcribed ad verbatim. Analysis of qualitative data was facilitated via MAXQDA software.
Timing	The study took place from October 2020 until December 2021.
Data exclusions	No data were excluded.
Non-participation	Response rate was high = 88.79 %. There were no drop-out cases.
Randomization	The study design required no allocation of participants to any groups.

Reporting for specific materials, systems and methods

We require information from authors about some types of materials, experimental systems and methods used in many studies. Here, indicate whether each material, system or method listed is relevant to your study. If you are not sure if a list item applies to your research, read the appropriate section before selecting a response.

Materials & experimental systems

n/a	Involved in the study
☒	☐ Antibodies
☒	☐ Eukaryotic cell lines
☒	☐ Palaeontology and archaeology
☒	☐ Animals and other organisms
☒	☐ Clinical data
☒	☐ Dual use research of concern

Methods

n/a	Involved in the study
☒	☐ ChIP-seq
☒	☐ Flow cytometry
☒	☐ MRI-based neuroimaging
