# Supplementary material for: Solving the explainable AI conundrum by bridging clinicians’ needs and developers’ goals
Source: NPJ Digit Med. 2023 May 22;6:94. doi: 10.1038/s41746-023-00837-4 (PMC10202353; doi:10.1038/s41746-023-00837-4)
Supplement: Supplementary file 2 — Supplementary Information [file 41746_2023_837_MOESM2_ESM.pdf]

Supplementary Information

Solving the Explainable AI Conundrum by Bridging the Gap Between Clinicians' Needs and Developers' Goals

Bienefeld, Nadine[1]*; Boss, Jens Michael[2]; Lüthy, Rahel[3]; Brodbeck, Dominique[3]; Azzati, Jan[3]; Blaser, Mirco[3]; Willms, Jan[2]; Keller, Emanuela[2]

[1] Department of Management, Technology, and Economics, ETH Zurich, Zurich, Switzerland
[2] Neurocritical Care Unit, Department of Neurosurgery and Institute of Intensive Care Medicine, Clinical Neuroscience Center, University Hospital Zurich and University of Zurich, Zurich, Switzerland
[3] Institute for Medical Engineering and Medical Informatics, School of Life Sciences FHNW, Muttenz, Switzerland

*Correspondence:
Bienefeld, Nadine
n.bienefeld@gmail.com

Supplementary Methods

Survey Part A **p. 2**

Survey Part B **p. 3**

Survey Part C **p. 3**

Supplementary Figures

Supplementary Figure 1 **p. 4**

Supplementary Figure 2 **p. 4**

Supplementary Figure 3 **p. 5**

Supplementary Tables

Supplementary Table 1 **p.6-7**

Supplementary References **p. 8**

Supplementary Methods

Survey instrument

PART A: Scenario-based questions

Patient Scenario 1:
You are responsible for a patient with aneurysmal subarachnoid hemorrhage (ruptured ACOM aneurysm managed by coiling). It is day 8 post hemorrhage. The patient has a GCS of 12, is partially agitated, and is manageable.

Question 1.
What would you do to assess this patient's risk for DCI? Please select one or multiple options below:

- Assess neurological status
- Check laboratory values (WBC, IL-6, CRP, PCT)
- Check blood flow velocity (Doppler)
- Check blood gases (current & over time)
- Check blood pressure & heart rate
- Check SpO2/paO2
- Check body temperature
- Check fluid balance
- Check laboratory values (GOT, GPT, Bili, Creatinine)
- Check ECG (electrocardiogram)
- Check CVP (central venous pressure

Patient Scenario 2:
As time progresses, the patient becomes sleepier, complains about a headache, and develops a fever. You can now use the DCIP application to support your assessment of this patient's secondary ischemia risk. The DCIP consists of a machine learning algorithm that estimates the patient's risk of secondary ischemia based on a multitude of parameters measured in each patient (e.g., monitoring data, laboratory values, and blood gases).

The DCIP indicates that the risk of secondary ischemia has increased from 60% to 90% in this patient.

Question 2.
Based on the information received from the DCIP, what would you do to assess the risk of DCI in this patient? Please select one or multiple options below:

- Assess neurological status
- Order CT with perfusion & angiogram
- Call for help from a peer (nurse or resident)
- Call the attending
- Check blood flow velocity (Doppler)
- Order new blood gas analysis
- Check all values from DCIP myself
- Optimize ventilation
- I would disregard the DCIP

Question 3.
Which factors would help you build trust in the DCIP? Please select one or multiple options below:

- I would trust the DCIP if I can understand how it works.
- I would trust the DCIP if it is highly reliable.
- I would trust the DCIP if I see that it works well in practice.
- I would trust the DCIP if it makes my work easier / faster.
- I would trust the DCIP if my colleagues (other nurses or physicians) trust the DCIP.
- I would trust the DCIP if it is officially certified as a medical device.
- I would not trust the DCIP in principle.

PART B: User Experience (UX)-related questions

Question 4.
How would you want to be alerted by the DCIP in case of a high estimated risk? Please select one option below:

- Audio and visual
- Visual only
- Audio only
- No alarms

Question 5.
Where should the DCIP be located so that it is easily accessible for you? Please select one or multiple options below:

- DCIP installed by patient bedside
- DCIP installed by the central patient monitoring station
- Remote access (via tablet or office computers)

PART C: Questions based on the Unified Theory of Technology Acceptance (UTATU)[1]

Question 6.
Performance expectancy (7-point Likert scale, 1 = strongly disagree to 7 = strongly agree)
I think the DCIP system will be useful in my job.
I think using the DCIP will improve the outcomes of my work.

Question 7.
Effort expectancy (7-point Likert scale, 1 = strongly disagree to 7 = strongly agree)
I think it will be easy for me to become skillful at using the DCIP system.
I think learning to operate the DCIP will be easy for me.

Question 8.
Intention to use (7-point Likert scale, 1 = strongly disagree to 7 = strongly agree)
I intend to use the DCIP as soon as it becomes available.
I will use the DCIP once it is introduced in the N-ICU.

Supplementary Figure 1: Frequencies (in %) of combined answers (physicians & nurses) for survey Q 4 regarding the DCIP alarm modalities.

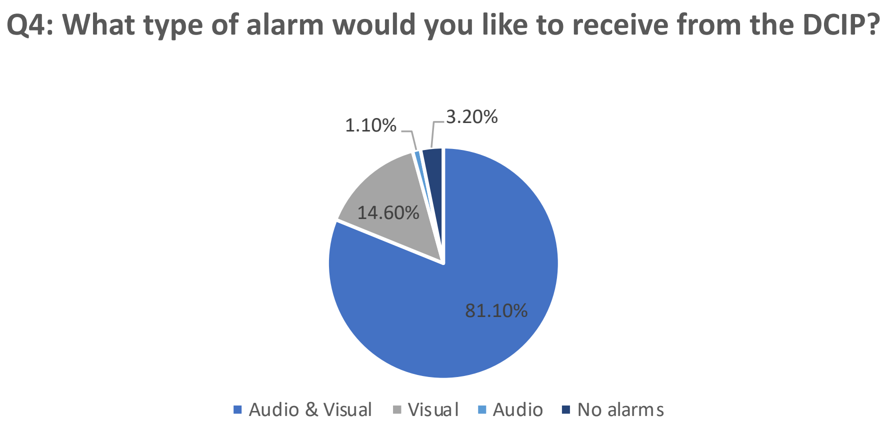

Supplementary Figure 2: Frequencies (in %) of answers for survey Q 5 regarding the DCIP location preference.

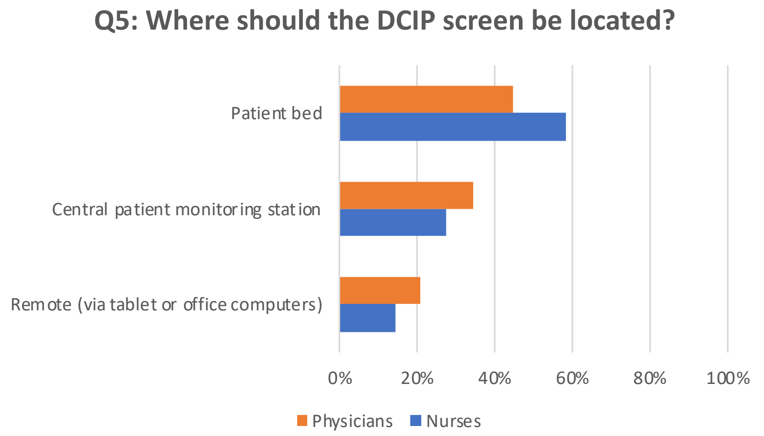

Note: Responses from physicians are displayed in orange and from nurses in blue (answers are sorted by role = physician).

Supplementary Figure 3. Data Structure based on the Gioia Methodology[2]

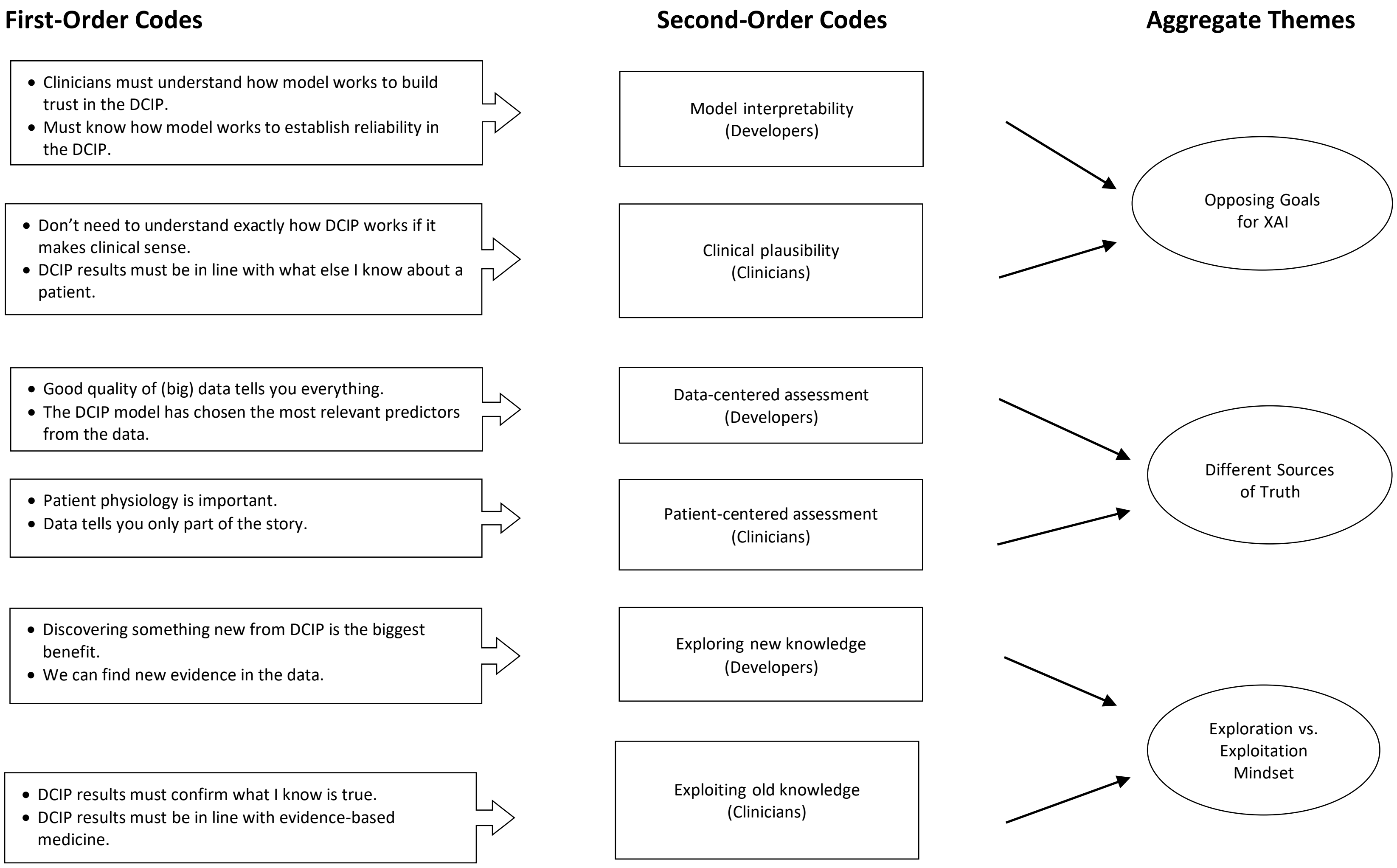

Supplementary Table 1. Selected Codes Generated from the Focus Group and Interviews Using Grounded Theory[3]

Code	Description of Code	Example Quotation
**Theme 1: Opposing Goals for XAI**		
Model Interpretability (Developers)	Describes how developers aimed at increasing the interpretability of the DCIP model by providing information such as static and dynamic contributor loadings, past trend data, or Shapley values.	"We use these Shapley values [referring to Figure 3, C 2 and E 5] to try and make the model explainable. These values can explain the local model, that is to explain why [the model] comes up with a certain decision at a specific point in time. To me, these explanations make intuitive sense. But sometimes it's difficult, as some scores seem to cancel each other out, and I don't know how clinicians are able to interpret this. On the other hand, if you ask them how they come up with a decision, I'm sure it's just as complicated." (Data scientists, 99)
Clinical Plausibility (Clinicians)	Describes how understanding the DCIP model itself was of limited interest to clinicians. Instead, clinicians wanted to understand the clinical plausibility of the included parameters and output data within the clinical context.	"I don't really need to know how the algorithm works. What I need to know is when [the DCIP] tells me the risk [of an upcoming DCI] is rising, why it is rising. That is which factors and which values do I need to look at and why". (Resident physician, 98)<br><br>"All I need to understand is why the risk for a particular patient to develop a DCI has changed over time. To be able to see what these parameters are and if they make sense clinically [referring to Figure 3, E 5]." (Attending physician, 96)
**Theme 2: Different Sources of Truth**		
Data-centered assessment (Developers)	Describes how ML-developers thought that the ground truth was included in the quantifiable patient data that was included in the DCIP model.	"It's all there in the model. [The data scientists] have selected all the parameters that best differentiate between patients who developed a DCI in the past and those that haven't. This includes the dynamic parameters over time such as blood gas values as well as the static parameters such as BMI or patients' age. And that's the power of the model to predict a DCI based on what the model has learned from all this data, to come up with that probability." (Product designer, 102)

Patient-centered assessment (Clinicians)	Describes how clinicians thought that the ground truth could only be found by combining quantifiable with non-quantifiable (e.g., information from the physical examination) patient data.	"I totally miss the information from the clinical assessment here [referring to the Shapley values of static and dynamic contributors Figure 3 C 2 and E 5]. A patient is more than just its data. For instance, it is crucial to know, if there is a change in consciousness or if the patient has developed paralysis somewhere. Sometimes, patients are able to talk, and all of a sudden, this changes. These are important pieces of the puzzle one should not ignore." (Attending physician, 103)
**Theme 3: Exploration vs. Exploitation Mindset**		
Exploring new knowledge (Developers)	Describes how ML-developers considered the possibility to learn and discover something new as the major benefit for ML-based predictions.	"We [ML-developers] always think, 'wow, look at what we can find in the data, that's amazing!' We always want to better understand 'what is the meaning of this particular pattern in the data', or 'why do these parameters combine in this or that way in the model'. But I acknowledge that others might not have an equally big interest in analyzing and learning from the data. For clinicians, who must do their job and make high-risk decisions based on [the system], it is probably safer to rely on what they already know. Not like us who can play around with the data for as long as we like knowing that nobody dies as a result of it." (Product designer, 102).
Exploiting old knowledge (Clinicians)	Describes how clinicians relied on established knowledge from evidence-based medicine to compare against and build trust in the DCIP model predictions.	"If the algorithm keeps showing me a new biomarker for which I have no clue that it has an influence on DCI [referring to Figure 3, E 5], it makes me wonder 'is [the system] just spitting out utter nonsense or did we [clinicians] just not know about this?'. If so, studies should look at this biomarker [e.g., Creatinine] and test if it really has something to do with DCI. Then one might declare the algorithm detected a new biomarker. But until then, I cannot trust it because there is no evidence from clinical studies." (Attending physician, 109).<br><br>"If the algorithm tells me, the probability [for a particular patient to develop a DCI] is 70% [referring to the overall risk score of the DCIP combining static and dynamic contributors, Figure 3, B 1] and I look at the patient and the neuromonitoring etc. and I don't see anything abnormal, I don't think I would trust [the system]. I might be alert and look more closely at all the information and make my own clinical assessment of the patient again but to take it at face value, no, it would take a long time to have this kind of trust in the system." (Attending physician, 105)

Supplementary References

1. Venkatesh, V., Sykes, T. A. & Zhang, X. 'Just What the Doctor Ordered': A Revised UTAUT for EMR System Adoption and Use by Doctors. in *2011 44th Hawaii International Conference on System Sciences* 1–10 (2011). doi:10.1109/HICSS.2011.1.
2. Gioia, D. A., Corley, K. G. & Hamilton, A. L. Seeking Qualitative Rigor in Inductive Research: Notes on the Gioia Methodology. *Organ. Res. Methods* **16**, 15–31 (2013).
3. Glaser, B. G. & Strauss, A. L. *Discovery of Grounded Theory: Strategies for Qualitative Research*. (Routledge, 2017).
